# Pro-death signaling of cytoprotective heat shock factor 1: upregulation of NOXA leading to apoptosis in heat-sensitive cells

**DOI:** 10.1038/s41418-020-0501-8

**Published:** 2020-01-29

**Authors:** Patryk Janus, Agnieszka Toma-Jonik, Natalia Vydra, Katarzyna Mrowiec, Joanna Korfanty, Marek Chadalski, Piotr Widłak, Karolina Dudek, Anna Paszek, Marek Rusin, Joanna Polańska, Wiesława Widłak

**Affiliations:** 1 Maria Sklodowska-Curie National Research Institute of Oncology, Gliwice Branch, Wybrzeże Armii Krajowej 15, 44-102 Gliwice, Poland; 2 Department of Data Science and Engineering, The Silesian University of Technology, Akademicka 16, 44-100 Gliwice, Poland

**Keywords:** Cell biology, Gene expression, Gene regulation

## Abstract

Heat shock can induce either cytoprotective mechanisms or cell death. We found that in certain human and mouse cells, including spermatocytes, activated heat shock factor 1 (HSF1) binds to sequences located in the intron(s) of the *PMAIP1 (NOXA)* gene and upregulates its expression which induces apoptosis. Such a mode of *PMAIP1* activation is not dependent on p53. Therefore, HSF1 not only can activate the expression of genes encoding cytoprotective heat shock proteins, which prevents apoptosis, but it can also positively regulate the proapoptotic *PMAIP1* gene, which facilitates cell death. This could be the primary cause of hyperthermia-induced elimination of heat-sensitive cells, yet other pro-death mechanisms might also be involved.

## Introduction

All organisms have developed mechanisms to protect themselves from damage during cellular stress (e.g., heat shock). Damaged cells are either repaired or eliminated to keep the integrity of the organism. Thus, either cytoprotection or programmed cell death can be induced in response to stress [1, 2]. Heat shock factor 1 (HSF1) is the primary transcription factor activated by different forms of proteotoxic stress. It becomes activated by forming trimers, which in turn bind specifically to heat shock elements (HSEs) throughout the genome. The HSE consensus sequence is a tandem array of at least three oppositely oriented “nGAAn” motifs or a degenerate version thereof. In most cells, HSF1 activation leads to accumulation of heat shock proteins (HSPs), molecular chaperones that help renature proteins unfolded during stress or direct them for degradation when repairing is impossible [3–5]. Moreover, HSPs prevent or suppress apoptosis by modulating both the mitochondrial or death receptor mediated apoptotic pathways and by interfering with caspase activation at several different levels [1, 6, 7]. Hence, the heat shock response (HSR) is defined as an inducible molecular response to a disruption of protein homeostasis which results in the elevated expression of cytoprotective genes to protect the proteome. Therefore, HSF1 is generally considered as a cytoprotective factor [8]. Nevertheless, despite the high degree of conservation of the HSR, different cells vary in their ability to induce HSPs synthesis and subsequently in sensitivity to proteotoxic agents. Moreover, some types of cells generally lack the HSR and are hypersensitive to elevated temperatures. In mammals, these include spermatocytes, certain neurons, preovulatory oocytes, and some stages of embryonic development, as well as T lymphocytes and certain cancer cell lines (especially of myeloid origin). Consequently, hyperthermia can cause infertility [9], neurological and cognitive dysfunction [10], embryonic death, abortion, growth retardation and developmental defects [11], while heat shock-induced T-cell apoptosis can be implicated in immune deficiency [12]. However, the molecular mechanisms involved in switching from pro-survival to pro-death signaling in cells subjected to heat shock remain to be elucidated.

PMAIP1 (phorbol-12-myristate-13-acetate-induced protein 1), also known as NOXA (Latin for damage), or APR (Immediate-Early-Response Protein APR), is a proapoptotic member of the BCL-2 protein family. It belongs to the BH3-only protein class, which contain a single BH-domain. PMAIP1 acts as a proapoptotic sensitizer/de-repressor: it interacts specifically with MCL1 or BCL2A1 proteins and targets them for degradation, thus neutralizing their antiapoptotic activity. It has been demonstrated that ectopically expressed PMAIP1 leads to the activation of caspase-9 and apoptosis in HeLa cells, while blocking endogenous PMAIP1 induction suppresses apoptosis [13]. In addition to its role in apoptosis, PMAIP1 also regulates diverse cellular functions in autophagic cell death and metabolism [14, 15]. Furthermore, it has been shown that PMAIP1 is a regulator of mitosis during early embryogenesis in zebrafish, while later in development the protein is a key mediator of apoptosis. Consequently, alterations in the expression of zebrafish *pmaip1* result in strong embryonic developmental defects [16]. *Pmaip1* is a p53-responsive gene and its encoded protein is involved in p53-dependent apoptosis triggered by irradiation and DNA damage [13]. However in some tissues, *Pmaip1* induction can occur in a p53-independent manner in response to other apoptotic stimuli, like hypoxia, proteasome inhibition, estrogen, glucose stress, etc. Thus, *Pmaip1* gene expression is regulated by multiple factors, including p73 (p53 homolog) [17], HIF1α [18], E2F1 [19], Brn3A/POU4F1 [20], c-Myc [21], ATF3, ATF4 [22], KLF6 and SP1 [23], ERα [24], FoxO1 [25], IRF-3 and NF-κB [26]. In addition, it is regulated posttranscriptionally by miR-200c, which represses both its basal and induced expression in response to various stimuli [27]. In the present study, we describe yet another mechanism of *Pmaip1* regulation and we partially disclose a mechanism of the pro-death response to heat shock. We show that following heat shock the *Pmaip1* gene becomes directly induced by HSF1 leading to apoptosis in heat-sensitive cells. However, some other mechanisms responsible for cell elimination are also activated, since PMAIP1 deficiency does not fully protect cells from heat-induced death.

## Materials and methods

### Animals, isolation of spermatocytes, and cell culture

Adult (10–16 weeks old), inbred FVB/N male mice were used for spermatocyte isolations and heat shock treatments. We also employed juvenile wild-type and transgenic males (three males for each experimental point) expressing a mutated, constitutively active transcriptionally competent form of HSF1 specifically in spermatogenic cells [28]. Spermatocytes were isolated by unit gravity sedimentation in linear BSA gradients as described earlier [29]. Mouse HECa10 endothelial cells of peripheral lymph nodes [30] (provided by Dr D. Duś, Institute of Immunology and Experimental Therapy, Wrocław, Poland) were grown in RPMI medium (Merck KGaA, Darmstadt, Germany) supplemented with 10% (v/v) heat-inactivated FBS (ICN Pharmaceuticals Inc, Costa Mesa, California, USA) and 40 µg/ml gentamicin sulfate (KRKA d.d., Novo Mesto, Slovenia). Human cell lines: 1205Lu melanoma, and HCT116 colon cancer were obtained from American Type Cell Culture Collection and cultured according to the supplier’s recommendations. Recombinant variant of HCT116 in which both *TP53* gene alleles were inactivated was received from the Dr. Bert Vogelstein group [31]. RKO colon carcinoma and RKO variant cells stably transfected with human papillomavirus E6 protein gene (RKO-E6) were a generous gift from Dr M. B. Kastan [32]. Cells were routinely tested for mycoplasma contamination. The animal experiments were carried out according to Polish legislation, and were approved by the Local Committee of Ethics and Animal Experimentation at the Medical University of Silesia in Katowice, Poland (Decisions No 82/2009 and No 129/2014) and by the Institutional Animal Care Policy of the Maria Skłodowska-Curie Institute—Oncology Center (Gliwice, Poland).

### Heat shock and drugs treatment

Whole-body heat treatment was performed in vivo in a water bath at 43 °C for 30 min as described earlier [33]. For each experimental point, three males were used. Animals were randomly divided between experimental groups. For ChIP experiments, equal volumes of CO_2_ saturated, preheated media (to 53 or 60 °C) were added to the spermatogenic cells suspensions, which immediately raised their temperature from 32 °C (physiological temperature) to 38 or 43 °C, respectively [29]. For somatic cells, media were preheated to 55 °C allowing the temperature to raise from 37 to 43 °C. Tubes were submerged in a water bath at the appropriate temperature for an additional 15 min. For transcriptional studies, suspensions of isolated spermatocytes or logarithmically growing adherent cells were heat shocked by placing them in a water bath at a temperature of 43 °C for 1 h (or for indicated time). The cells were allowed to recover for indicated time in a CO_2_ incubator at 32 or 37 °C, respectively. The growth media were not replaced either before or after heat shock. Other cells treatments included incubation with 5 µM camptothecin (CPT) or 32 nM bortezomib for indicated time.

### Chromatin immunoprecipitation

Data on HSF1 binding to *Pmaip1* assessed in genome-wide ChIP-Seq analysis were extracted from the dataset available on Gene Expression Omnibus, accession no. GSE56735 [34]. The ChIP assay was carried out according to the protocol of a ChIP kit from Upstate Biotechnology (Lake Placid, NY, USA) using protein A-sepharose beads (GE Healthcare Europe GmbH, Freiburg, Germany) or according to the Dynabeads™ Protein A from Life Technologies protocol (Thermo Fisher Scientific, Waltham, MA, USA), or according to the protocol from the iDeal ChIP-Seq Kit for Transcription Factors (Diagenode, Denville, NJ, USA). Standard crosslinking procedure was used. For each IP reaction, 30 µg of chromatin sonicated to 100–500 bp fragments and 3 µg of rabbit anti-HSF1 polyclonal antibody (ADI-SPA-901, Enzo Life Sciences, Farmingdale, NY, USA) were used. For negative controls chromatin samples were processed with IgG from rabbit serum (Merck KGaA) or without antibody. Immunoprecipitated DNA was dissolved into 20 µl of H_2_O and analyzed by PCR/qPCR using 1 µl as a template and 40 PCR cycles. In “Input” sample, 0.003–0.006% (ChIP-PCR) or 1% (ChIP-qPCR) of a total material used for the ChIP assay served as a template.

### RNA isolation, cDNA synthesis, and PCR/qPCR

Total RNA was isolated using the GeneMATRIX Universal RNA Purification Kit (Eurx, Gdańsk, Poland) or the Direct-ZolTM RNA MiniPrep Kit (Zymo Research, Irvine, CA, USA), digested with DNase I (Worthington Biochemical Corporation, Lakewood, NJ, USA), converted into cDNA, and used for RT-PCR as described [29]. Quantitative PCR was performed using a BioRad C1000 TouchTM thermocycler connected to the head CFX-96. Each reaction was performed at least in triplicates and contained: 1× PCR Master Mix SYBRGreen (A&A Biotechnology, Gdynia, Poland), 200 nM of each primer, and cDNA template (equivalent of 10 ng of transcribed RNA). Expression levels (RT-qPCR) were normalized against *GAPDH* and *HNRNPK*. The set of delta-Cq replicates (Cq values for each sample normalized against geometric mean of the reference genes) for control and tested samples were used for statistical test and estimation of the *p*-value. Shown are median, maximum and minimum values of a fold-change versus untreated control. Chromatin occupancy (ChIP-qPCR) for each sample was calculated against corresponding input value. The set of delta-Cq replicates for control and tested sample were used for statistical test as described above. Experiments were repeated 2–4 times. The primers used in these assays are described in Table S1.

### TUNEL assay and immunofluorescence (IF)

Reactions were performed on sections (6 µm) of formalin-fixed (10% in PBS, overnight at 4 °C) and paraffin-embedded mouse organs. An antigen retrieval step in 0.01 M citrate buffer pH 6.0 was performed before procedures. Apoptotic cells with DNA breaks were detected using the In Situ Cell Death Detection Kit, TMR red (Cat. No. 12 156 792 910, Roche, Indianapolis, IN, USA) according to the supplier’s protocol. IF imaging was performed using a primary antibodies: anti-PMAIP1 (1:200; PRS2437, Merck KGaA; for specificity tests, see Supplementary Fig. S1) or anti-HSF1 (1:200; ADI-SPA-901, Enzo Life Sciences) and Alexa Fluor 647 Tyramide SuperBoost Kit goat anti-rabbit IgG (B40926, Invitrogen, Waltham, MA, USA) according to the user guide. TUNEL and IF were executed separately or immediately after TUNEL reaction IF was implemented. Finally, the DNA was stained with DAPI. Images were captured at ×630 magnification using Zeiss Axio Imager.M2 fluorescent microscope with AxioCam camera and AxioVision (Rel.4.8) imaging system (Alexa Fluor 647 was pseudo-colored in green). Negative controls were performed in parallel on serial slides for specific labeling by omitting the primary antibody or by the peptide competition. For the latter one, the primary antibody in working concentration was preincubated for 1 h at room temperature with a 5-time excess of the synthetic peptide antigen (SBP2437, Merck KGaA).

### Protein extraction and western blotting

Cellular proteins were extracted using a Radioimmunoprecipitation Assay buffer consisting of 1× PBS, 1% NP-40, 0.5% SDC, 0.1% SDS, 1 mM PMSF, 50 mM NaF, and protease inhibitors cocktail (Roche). Proteins (10–50 µg) were separated on 10 or 18% SDS-PAGE gels and electrotransferred to 0.22-µm or 0.45-µm pore nitrocellulose filters or onto a PVDF membrane (Merck Millipore, Burlington, MA, USA). Primary antibodies used were: anti-PMAIP1 (1:1,000; PRS2437, Merck KGaA, or PA5-19977, Thermo Fisher Scientific; immunogen sequence: PGRKARRNAPVNPTRAE; for specificity tests, see Supplementary. Fig. S1) and corresponding blocking peptide (SBP2437, Merck KGaA), anti-HSPA1 (1:2,000; ADI-SPA-810), and anti-HSF1 (1:1,000; ADI-SPA-901) both from Enzo Life Sciences, anti-caspase-3 (1:3,000; #14220), anti-cleaved caspase-3 (1:1,000; #9664), anti-caspase-7 (1:3,000; #12827), and anti-cleaved caspase-7 (1:1,000; #8438), and anti-PARP1 (1:2,000; #9542) from Cell Signaling Technology (Danvers, MA, USA). As a loading control, anti-actin (1:10,000; MAB1501, Millipore) was used. The primary antibody was detected by an appropriate secondary antibody conjugated with horseradish peroxidase (1:1,000–1:5,000) and visualized by WesternBright ECL or WesternBright Sirius kits (Advansta, Menlo Park, CA, USA). Imaging was performed on x-ray film or in G:BOX chemiluminescence imaging system (Syngene, Frederick, MD, USA). To test anti-PMAIP1 antibody specificity, the antibody in working concentration was preincubated for 1 h at room temperature with a 5-time excess of the blocking peptide. The experiments were repeated at least in triplicate and blots were subjected to densitometric analyses using Image Studio Lite software to calculate relative protein expression after normalization with loading controls (statistical significance of differences was calculated using *T*-test).

### Cloning and mutagenesis of *Pmaip1* sequences

Part of the second intron (1–1709 bp from the whole 2300 bp) of the mouse *Pmaip1* gene, containing HSE, was amplified by PCR on DNA template. *SalI* and *BamHI* sites were introduced into PCR primers and the sequence was inserted as a enhancer below SV40 late poly(A) signal in pGL3-Promoter vector (Promega, Madison, WI, USA) encoding the firefly luciferase under the control of SV40 promoter. HSE, i.e., A**GAA**AG**TTC**TC**GAA**G, was mutated to AtA*AAGcTt*TCaAAG (*HindIII* site was introduced) using the GENEART® Site-Directed Mutagenesis System (Invitrogen) according to the manufacturer’s protocol. The DNA sequence of the recombinant plasmids was confirmed by sequencing (Genomed, Warszawa, Poland).

### Dual-luciferase reporter assay

We used previously established 1205Lu melanoma cell lines: stably expressing the active form of HSF1 (aHSF1) and corresponding control with the empty vector (Neo) [35]. Cells were seeded into 96-well plates (1 × 10^4^/well) and transfected the next day with TurboFect (Thermo Fisher Scientific) according to the instructions provided by the manufacturer. A total of 104 ng plasmid DNA/well was transfected, including experimental pGL3-Promoter vector and its derivatives (containing the firefly luciferase gene) and co-reporter pRL-TK plasmid (containing the *Renilla* luciferase gene) (Promega) in ratio 50:1. As a positive control we used 1324 bp *BglII* and *HindIII* fragment of the human *HSPA7* promoter re-cloned from p2500-CAT vector [36] into corresponding sites in pGL3-Basic vector (Promega). Transfections were done in triplicate. The cells were lysed 24–48 h after transfection in the Passive Lysis Buffer and the cell lysates were assayed using the Dual-Luciferase Reporter assay kit (Promega) according to the manufacturer’s instructions. Luminescence was detected using a Synergy 2 microtiter plate reader (BioTek Instruments, Winooski, VT, USA). Relative luciferase activity was determined by normalizing the activity of the firefly luciferase activity (*F* value) against the *Renilla* luciferase activity (*R* value). The *F*/*R* value was calculated respectively and an average value was obtained as the recombinant plasmid activity. Then, the activity in cells with aHSF1 was compared with the activity in control Neo cells. Each experiment was performed three times.

### CRISPR/Cas9 genome editing of the *Pmaip1* sequences

In order to remove the perfect HSE from the second intron of the mouse *Pmaip1* gene (Gene ID: 58801), we utilized Guide-it™ CRISPR/Cas9 System (Red) from Clontech (Saint-Germain-en-Laye, France). Single guide RNA sequences (sgRNAs) were designed using the CRISPR Design webtool (http://crispr.mit.edu/). Three sequences were selected and tested for a cleavage efficiency using Guide-it sgRNA In Vitro Transcription and Screening System according to the Clontech user manual. Two chosen sgRNAs were: “left” 5′-ACTTTCTTACAGTGTTCCTA (on minus strand) and “right” 5′-CAGCAGCTTGCATGTTAGTC (on plus strand). They targeted the sequences (underlined below) immediately adjacent to an ***NGG*** Protospacer Adjacent Motif (PAM) sequences (italic) around the HSE (A**GAA**AG**TTC**TC**GAA**G) on chromosome 18, position from 66461075 to 66461128: ***CCT***TAGGAACACTGTAA**GAA**AG**TTC**TC**GAA**GCAGCAGCTTGCATGTTAGTC***AGG****.* The “right” sgRNA was cloned into pGuide-it-tdTomato vector (Clontech) which enabled simultaneous expression of Cas9 and the fluorescent protein tdTomato. The “left” sgRNA together with U6 promoter, guide RNA scaffold, and termination signal was synthesized as a GeneArt_Strings_DNA_Fragment (Thermo Fisher Scientific) according to Mali et al. [37] and introduced into vector containing EGFP under control of SV40 promoter. The DNA sequence of the recombinant plasmids was confirmed by sequencing (Genomed). Subconfluent HECa10 cells were transiently transfected with 10 µg of each plasmid coding for sgRNAs and K2 transfection reagent (Biontex, Munich, Germany) in serum-free medium for 6 h according to the manufacturer’s instructions. After 36 h, transfected cells were sorted by flow cytometry (FACS Canto-Becton Dickinson, Franklin Lakes, NJ, USA). Cells positive for red and green fluorescence were collected, seeded on plate, and cultivated until they reached 50% confluency. Single HECa10 clones were obtained by limiting dilution on a 96-well plate. Individual wells were examined by microscopy to exclude polyclonal cell lines. To assess the deletion of the HSE fragment the whole genomic DNA was isolated (Genomic Mini Kit, AA Biotechnology, Gdynia, Poland) from every of monoclonal cell line. The 201 bp long DNA fragment around the HSE sequence was amplified by PCR with specific primer pair: F: ATGAGGAGCCCAAGCCCAAC, R: ACCCCTGTGGAGGTGAGCAA. PCR products were resolved on agarose gel, purified with magnetic beads (MAGBIO Genomics, Gaithersburg, MD, USA) and subjected to Sanger sequencing (Genomed) using the primer F. In the clone with hemideletion (1/2HSE), one allele was intact while the sequence between PAMs in the second allele was ***CCT***CAT/Δ38bp/GTTAGTC***GGG***.

### HSF1 functional knockout

In order to remove the human *HSF1* gene, Edit-R Human HSF1 (3297) crRNA (Table S2) from Dharmacon (Lafayette, CO, USA), Edit-R tracrRNA, and eSpCas9-GFP protein (#ECAS9GFPPR, Merck KGaA) were introduced into RKO cells using Viromer® CRISPR (Lipocalyx GmbH, Halle (Saale), Germany) according to the manual provided by the producer. Single clones were obtained by limiting dilution on a 96-well plate. An efficiency of the HSF1 knockout was monitored by western blot and confirmed by sequencing (Genomed). Six individual clones with the *HSF1* knockout and six pooled control clones were analyzed for the HSR.

### PMAIP1 functional knockout

In order to knockout the mouse *Pmaip1* gene in HECa10 cells, we utilized CRISPR/Cas9 system from Dharmacon which included: Edit-R synthetic crRNAs (Table S2), Edit-R tracrRNA, and Edit-R hCMV-PuroR-Cas9 Expression Plasmid. As a negative control, Edit-R crRNA nontargeting control #1 (U-007501-01-05) was used. HECa10 cells were seeded on 12 well plates 1 day before transfection. Subconfluent cells were transiently transfected with DharmaFECT Duo (6 µg/ml) and crRNAs (50 nM each), tracrRNA (150 nM), and hCMV-PuroR-Cas9 plasmid (2 µg/ml) in a 1 ml of serum-free RPMI medium. After 48 h, the medium was replaced with fresh one containing FBS. Selection medium with puromycin (4 µg/ml) was added after 24 h and cells were cultivated for 48 h. Single clones were obtained by limiting dilution on a 96-well plate. To assess the deletion of the *Pmaip1* gene, the whole genomic DNA was isolated (Genomic Mini Kit, AA Biotechnology) from each single clone and 2542 bp long fragment was amplified with specific primer pair: 21Pmaip1F: CCTACTGAAG CTCGGTGCGT CT and intr2Pmaip1R: 5′- ACCCCTGTGG AGGTGAGCAA. PCR products were analyzed on agarose gel, purified on the DNA Clean & Concentrator columns (Zymo Research), and subjected to Sanger sequencing (Genomed) using the sequencing primers: 21Pmaip1F and intr2Pmaip1R. The PMAIP1 protein level was additionally assessed by western blot after CPT stimulation (5 µM for 6 h). Fifteen individual clones with a complete *Pmaip1* knockout or six control clones were pooled and used in further experiments.

### Cell death assay

Cells were seeded into 96-well plates (3 × 10^3^/well) 1 day before treatments. They were treated with heat shock, bortezomib, or CPT and the viability was analyzed on the Synergy 2 microtiter plate reader (BioTek Instruments) using the RealTime-Glo™ Annexin V Apoptosis and Necrosis Assay (Promega, # JA1011) (which allows continual monitoring of cell state) according to the manufacturer’s instructions. Three replicates were performed in four independent experiments.

### Statistical analysis

For each dataset the normality of distribution was assessed by the Jarque–Bera test and homogeneity of variances was verified by the *F*- or Bartlett’s tests depending on the subgroup number to provide optimal tools for statistical analysis; moreover, single outliers were identified by Dixon’s test, while multiple outliers—by the Iglewicz–Hoaglin test. For analysis of differences between compared groups the quality of mean values was verified by the ANOVA test with pairwise comparison done with Dunnett’s test. In case of non-Gaussian distribution, the Kruskal–Wallis ANOVA was applied for the verification of the hypothesis on the equality of medians with Dunn’s test for pairwise comparisons. *P* = 0.05 was selected as a statistical significance threshold.

## Results

### Heat shock-induced binding of HSF1 in the introns of the *Pmaip1* gene correlates with upregulation of its expression and enhanced apoptosis in heat-sensitive mouse cells

**Fig. 1:**
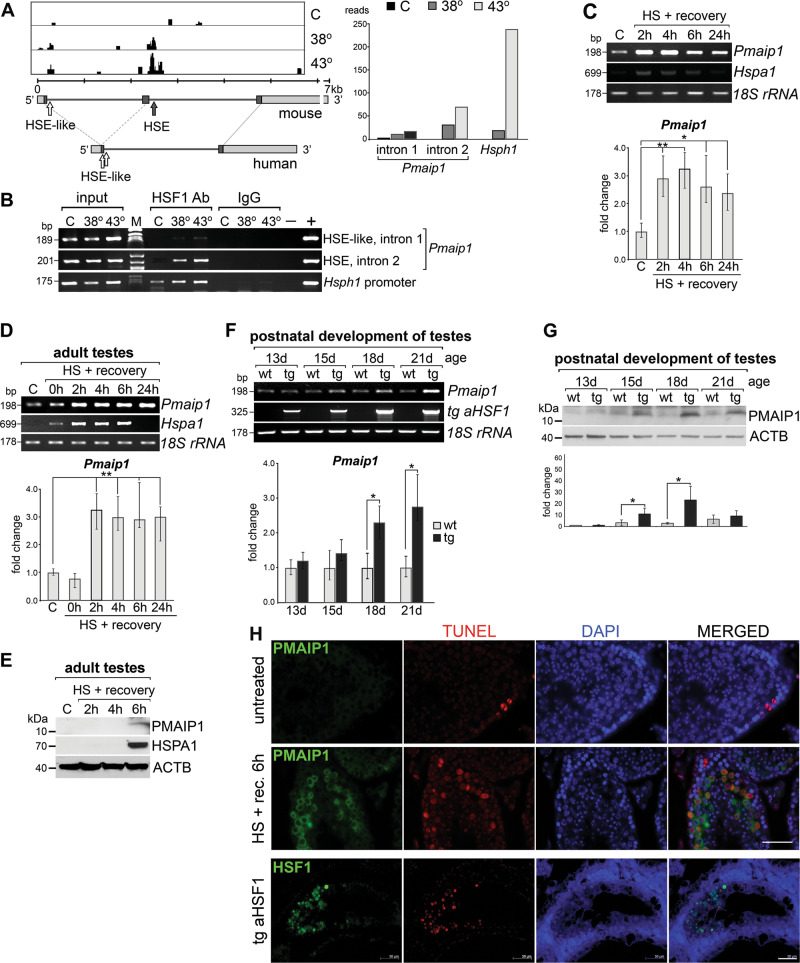
Heat shock-induced HSF1 binding in the introns of the *Pmaip1* gene correlates with upregulation of its expression and enhanced apoptosis in mouse spermatogenic cells. **a** Chromatin binding of HSF1 assessed by ChIP-Seq in isolated spermatocytes. Organization of mouse and human genes is shown below peaks of ChIP-Seq tags: bars—exons (darker bars—coding regions), lines—introns; corresponding start and stop codons are linked by light-gray dashed or solid lines, respectively; the positions of HSE or HSE-like motifs are indicated by the closed and open arrows, respectively. Right panel shows the magnitude of HSF1 binding in intronic HSE of the *Pmaip1* gene in comparison to *Hsph1* promoter based on data from ChIP-Seq extracted from GSE56735. **b** HSF1 binding in *Pmaip1* introns analyzed by ChIP-PCR in isolated spermatocytes. Binding to the *Hsph1* promoter is shown as a positive control. C control, physiological temperature of testes (32 °C); 38° and 43°, heat shock at 38 or 43 °C, respectively; M marker; − +, negative and positive PCR controls. **c** Induction of *Pmaip1* transcription assayed by RT-PCR and RT-qPCR in isolated spermatocytes after heat shock in vitro at 43 °C and **d** in testes of mice after heat shock in vivo. *18S rRNA* and *Hspa1* were used as transcript level controls for loading and the heat shock response, respectively; C control, HS heat shock. **e** Accumulation of PMAIP1 protein after heat shock in vivo in mouse testes demonstrated by western blot. ACTB and HSPA1 were used as controls for loading and the heat shock response, respectively. **f** Induction of *Pmaip1* transcription assayed by RT-PCR and RT-qPCR in testes of transgenic mice expressing constitutively active mutated HSF1 (*aHSF1*) during postnatal development; wt wild type, tg transgenic. Asterisks on the graphs indicate statistical significance of differences: *p < 0.05, **p < 0.001. **g** Accumulation of PMAIP1 in transgenic mouse testes demonstrated by western blot. ACTB was used as a control for loading. **h** Detection of PMAIP1 or HSF1 by immunofluorescence (green) and apoptotic DNA breaks (by TUNEL assay, red; DNA stained with DAPI, blue) in seminiferous tubules (stages IX–X) of untreated mice and after 6 h of recovery from heat shock in vivo (PMAIP1; upper panels) or the aHSF1 transgenic mouse (HSF1; bottom panel). Scale bar—50 µm.

Using a genome-wide ChIP-Seq approach we found that HSF1 became localized in the second intron of the *Pmaip1* gene in the chromatin of heat-shocked mouse spermatocytes [34]. This HSF1 binding occurred at a perfect HSE motif. We confirmed this binding by ChIP-PCR in isolated spermatocytes after either mild or strong heat shock at 38 or at 43 °C, respectively. Moreover, we also detected heat shock-induced occupancy of HSF1 in the first intron of the *Pmaip1* gene, where an HSE-like sequence is located (Fig. 1a, b). To determine whether HSF1 occupancy correlated with increased *Pmaip1* expression, we analyzed its transcription in isolated spermatocytes (Fig. 1c) and whole testes (Fig. 1d) from mice subjected to heat shock at 43 °C which showed an evident *Pmaip1* upregulation. Accumulation of the PMAIP1 protein after heat shock in mouse testes was observed as well (Fig. 1e). We also took advantage of a mouse strain that expressed a mutated, constitutively active form of HSF1 (aHSF1) specifically in spermatogenic cells [28, 38]. Notably, we found that *Pmaip1* gene transcription was upregulated at physiological temperature in the testes of these transgenic mice (Fig. 1f). The level of *Pmaip1* transcripts increased concomitantly with the accumulation of transgenic HSF1 during postnatal development. This was associated with increased levels of the PMAIP1 protein in the testes of transgenic mice (Fig. 1g), in which apparent HSF1-induced apoptosis of spermatocytes occurred [28]. Such upregulation of the *Pmaip1* expression correlated with the PMAIP1 protein accumulation specifically in spermatocytes and round spermatids and massive apoptosis observed in seminiferous tubules after heat shock (Fig. 1h, see Supplementary Fig. S2 for kinetics). IF analysis also showed a correlation between HSF1 accumulation and apoptotic cell death in the testes of transgenic mice (Fig. 1h, bottom panel). These results demonstrate collectively that increased occupancy of HSF1 at the HSE present in the second intron (and possibly to HSE-like sequences present in the first intron) of the *Pmaip1* gene directly correlates with an increase in the steady-state level of the *Pmaip1* gene’s transcripts in mouse spermatogenic cells.

Next, we determined if the *Pmaip1* expression could also be activated by heat shock in somatic cells and how it is associated with sensitivity of these cells to elevated temperatures. The highest degree of the *Pmaip1* activation (mRNA accumulation within 2 h of recovery from heat shock) was observed in the spleen, thymus, stomach, and small intestine. In contrast, the *Pmaip1* transcripts accumulated later on after heat shock in the kidney and were hardly detected in the liver (Supplementary Fig. S3A). Tissue-specific differences in the level of heat shock-induced PMAIP1 were confirmed at the protein level by western blot (Supplementary Fig. S3B). Furthermore, detection of apoptotic DNA breaks by TUNEL assay in organs of heat-shocked mice (Supplementary Fig. S4) showed correlation between *Pmaip1* activation and heat sensitivity. The number of apoptotic cells was increased substantially (versus untreated control) 2–6 h after heat shock in lymphoid tissues, like the thymus and spleen. The effects were less profound in the stomach (where single clusters of dying cells were found) and the intestine, while not visible in the kidney when early effects of heat shock (up to 24 h) were analyzed. Therefore, we hypothesized that different heat sensitivity of these organs depends on their ability to induce the *Pmaip1* gene expression following HSF1 activation in response to heat shock.

### HSF1 and HSEs present in the introns of *Pmaip1* gene are essential for the gene activation after heat shock

**Fig. 2:**
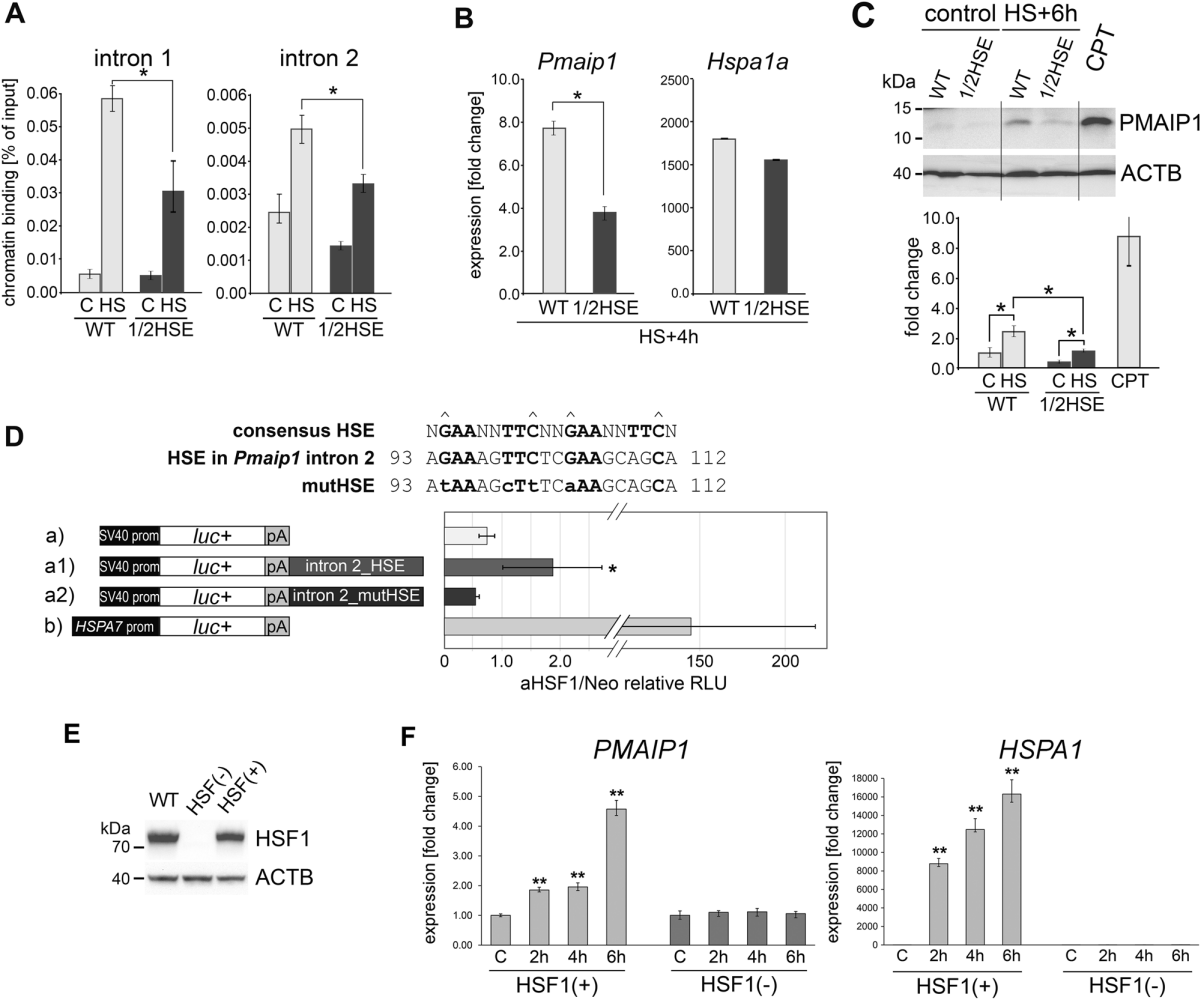
HSF1 and HSEs present in the introns of *Pmaip1* gene are essential for its activation after heat shock. **a** Heat shock-induced HSF1 binding in introns of *Pmaip1* analyzed by ChIP-qPCR in wild-type (WT) HECa10 cells and the clone with hemideletion (1/2HSE) of the perfect HSE in the second intron. **b** RT-qPCR assays of *Pmaip1* and *Hspa1a* genes transcript levels after heat shock (HS) in cells described in panel **a**. Fold changes in reference to untreated cells are shown. **c** PMAIP1 level in the same cells analyzed by western blot. CPT treatment served as positive control for PMAIP1 upregulation. ACTB is shown as a control for loading. Lower panel shows the representative results of densitometric analyses of western blots; *p < 0.05. **d** Relative luciferase activity in the human 1205Lu cells stably expressing constitutively active HSF1 (aHSF1) in relation to control cells with the empty vector (Neo). Cells were transiently transfected with: the pGL3-Promoter vector (a), its derivatives with the part of the second intron of the mouse *Pmaip1* gene acting as an enhancer, containing either wild-type (a1) or mutated HSE (a2), and the vector with the *HSPA7* promoter (b) used as a positive control. Sequences of wild-type HSE from the second intron of mouse *Pmaip1* (nucleotides 93–112 downstream from the exon2/intron2 boundary) and mutated HSE (mutHSE) are shown above the graph. Hats indicate the most essential G and C nucleotides in the HSE sequence. Presented are mean values and ± SD from three independent experiments (with three-five technical repeats each); *p < 0.05. **e** HSF1 protein levels detected by western blot documenting the complete HSF1 knockout (−) obtained in RKO cells by CRISPR/Cas9 editing. ACTB is shown as control for loading. **f** RT-qPCR assays of *PMAIP1* and *HSPA1A* transcript levels after heat shock treatment in HSF1(+) (mix of control clones) and HSF1(−) (one of six individual clones; the same result was obtained for all clones) RKO cells. **p < 0.001.

Among all tested mouse cell lines, the highest *Pmaip1* activation after heat shock was observed in HECa10 endothelial cells from peripheral lymph nodes (Supplementary Fig. S5A). We assumed that cells which activate the *Pmaip1* gene transcription could be more sensitive to heat-induced damage. Thus, we compared the extent of cell death induced by severe heat shock in mouse cell lines that either activate (HECa10) or do not activate (B16-F10) the *Pmaip1* gene transcription after heat shock (Supplementary Fig. S5B). Although increases in cell death occurred after heat shock in both tested cell lines, markedly higher increase in the number of dead cells was observed in HECa10 cells (Supplementary Fig. S5C). This cell line was used in further experiments as a heat-sensitive mouse somatic cell model. In HECa10 cells, binding of HSF1 was detected in the second intron (containing the perfect HSE) as well as in the first intron (containing HSE-like motif) of the *Pmaip1* gene (Fig. 2a and Supplementary Fig. S5D), but not in two HSE-like sequences in the promoter (not shown). No HSF1 binding was detected in B16-F10 cells (Fig. S5D). HSF1 binding correlated with increased *Pmaip1* transcription (Fig. 2b and Supplementary Fig. S5). It is noteworthy that other known HSF1-activating agents (i.e., bortezomib and sodium arsenite) also caused *Pmaip1* upregulation in HECa10 but not in B16-F10 cells (Supplementary Fig. S6). We then checked whether the human *PMAIP1* gene, which has a different structure compared with the mouse gene, could be similarly regulated by HSF1. The intron of the human *PMAIP1* gene contains an HSE-like motif, which is similar to the corresponding region in the first intron of the mouse gene (see Fig. 1a). We found HSF1 binding to this HSE motif (but not to HSE-like motif in exon 1) which correlated with the *PMAIP1* mRNA accumulation in different human cell lines treated with heat shock (1205Lu cell line which was further used in luciferase studies is shown as an example in Supplementary Fig S5B, D).

To test the importance of the perfect HSE located in the second intron of the mouse *Pmaip1* gene, we planned to mutate this HSE in the genome of HECa10 cells applying the CRISPR/Cas9 approach. Due to the lack of PAM (Protospacer Adjacent Motif) sequences directly in HSE, we intended to remove the whole HSE region using two flanking guide RNAs. However, after selection of individual clones only one clone (out of five which possessed short indels) showed hemideletion of HSE (i.e., deletion in one allele) that was confirmed by DNA sequencing. Nevertheless, this hemideletion of HSE resulted in visibly reduced heat shock-induced binding of HSF1 (interestingly, in both intronic HSEs, which suggested their cooperation in response to heat shock) (Fig. 2a) and reduced activation of *Pmaip1* when compared with not modified parental cells (Fig. 2b). This was associated with the lower PMAIP1 protein level (Fig. 2c). To further analyze the effect of the intronic HSE on gene expression, the luciferase model was implemented. We used a modified human 1205Lu cell line stably expressing a constitutively aHSF1 and the corresponding control cells with the empty vector (Neo; [35]) to check if the mouse *Pmaip1* intronic sequences containing the perfect HSE could work as an enhancer to increase the luciferase expression in the HSF1-dependent but heat shock-independent manner. The part of the second mouse *Pmaip1* intron was cloned as an enhancer in the pGL3-Promoter vector (with the firefly luciferase reporter gene under the control of SV40 promoter). We found that in cells stably expressing aHSF1, the HSE-containing region was able to enhance the luciferase expression above the level observed in control (Neo) cells. When we introduced mutation into HSE, the activity of the reporter gene was comparable to that from the control vector without the enhancer (Fig. 2d). This indicates that active HSF1 is able to stimulate the expression through the HSE-containing enhancer (originating from the mouse *Pmaip1* intron) in human cells, although effectiveness of the classical HSE-containing promoter (i.e., HSPA7 promoter used as a positive control) was apparently much higher. These differences reflected the degree of *Pmaip1* and *Hsp* genes activation following heat shock. Furthermore, we documented that knockout of the HSF1 in RKO cells (Fig. 2e) resulted in a complete inhibition of the *PMAIP1* activation after heat shock, similarly to the typical HSF1-dependent *HSPA1* genes (Fig. 2f). Presented data collectively indicates that HSF1 is indispensable for *Pmaip1/PMAIP1* activation after heat shock via binding to the intronic HSEs, although its interactions with other regulatory sequences cannot be excluded.

### Heat shock activates *PMAIP1* expression in the p53-independent manner

**Fig. 3:**
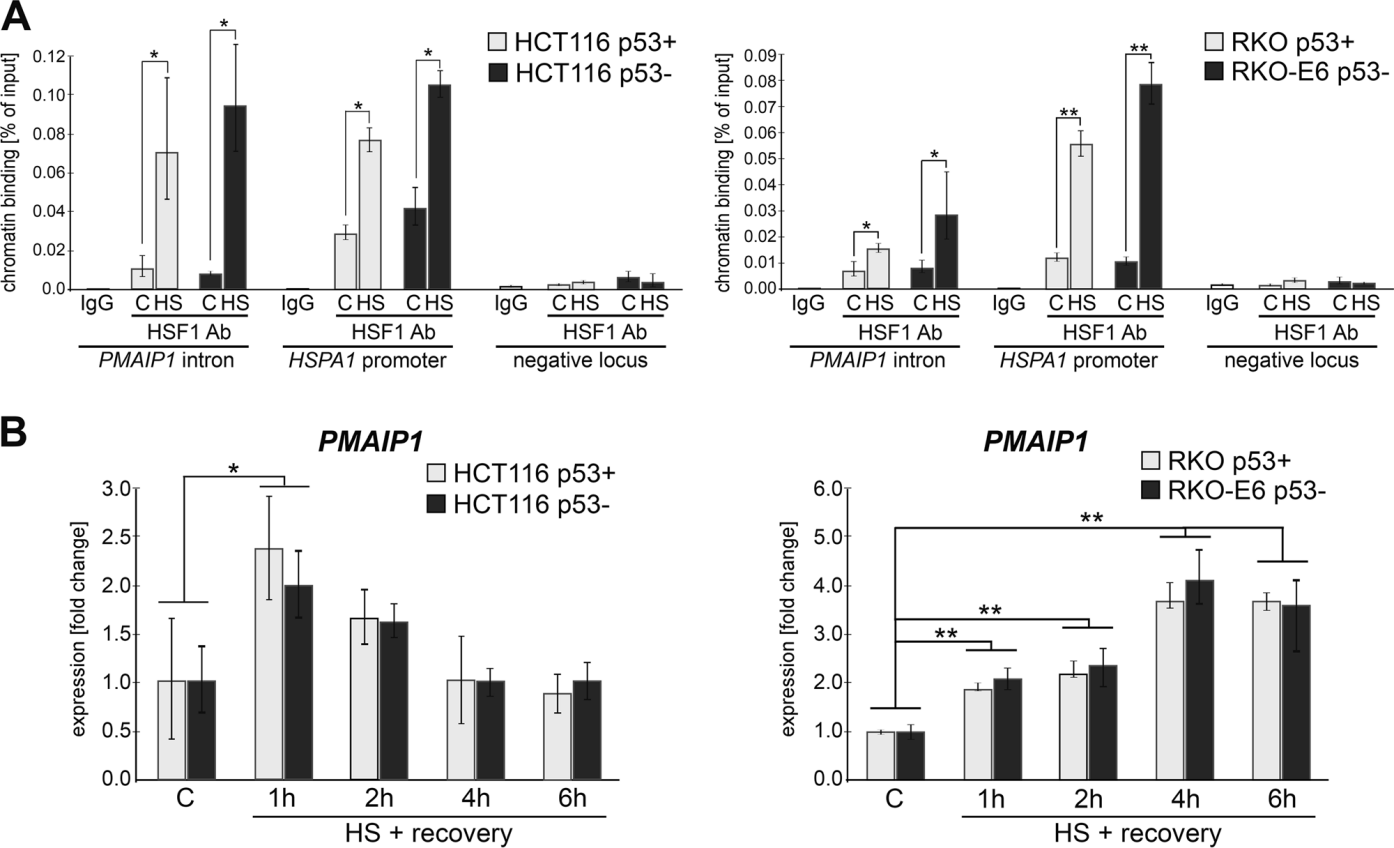
*PMAIP1* activation by heat shock is independent of p53. **a** HSF1 binding in *PMAIP1* intron analyzed by ChIP-qPCR in p53+ or p53− variants of HCT116 and RKO cells. Binding to the *HSPA1A* promoter and negative locus served as positive and negative controls, respectively. IgG, ChIP performed with IgG instead of HSF1. **b** *PMAIP1* transcript levels analyzed by RT-qPCR in p53+ or p53− variants of HCT116 and RKO cells; the experiment was repeated four times; *p < 0.05, **p < 0.001, the statistical significance of differences between control and test samples.

Since *PMAIP1* is a primary p53-responsive gene, we checked whether p53 is required for *PMAIP1* upregulation following heat shock. For this purpose, we compared human cells with different p53 status: HCT116 colon carcinoma cells with wild-type p53 and HCT116 variant with the *TP53* gene depletion due to its bi-allelic knockout [31] as well as RKO colon carcinoma cells (wild-type p53) and RKO variant (RKO-E6) that expresses the human papillomavirus E6 protein which binds to and inactivates p53 protein. All tested cell lines responded to heat shock by activation of HSF1 which was manifested by its increased binding to HSE in both *PMAIP1* intron and *HSPA1A* promoter (Fig. 3a). This was followed by increased expression of *PMAIP1* (Fig. 3b) and *HSPA1A* (not shown) in both cell variants. Most importantly, no difference in heat shock-induced activation of *PMAIP1* was observed between p53-proficient and p53-deficient cells. Hence, HSF1-mediated activation of *PMAIP1* gene after heat shock appears to be independent of p53.

### PMAIP1 deficiency reduces heat-induced cell death and delays the activation of caspases

**Fig. 4:**
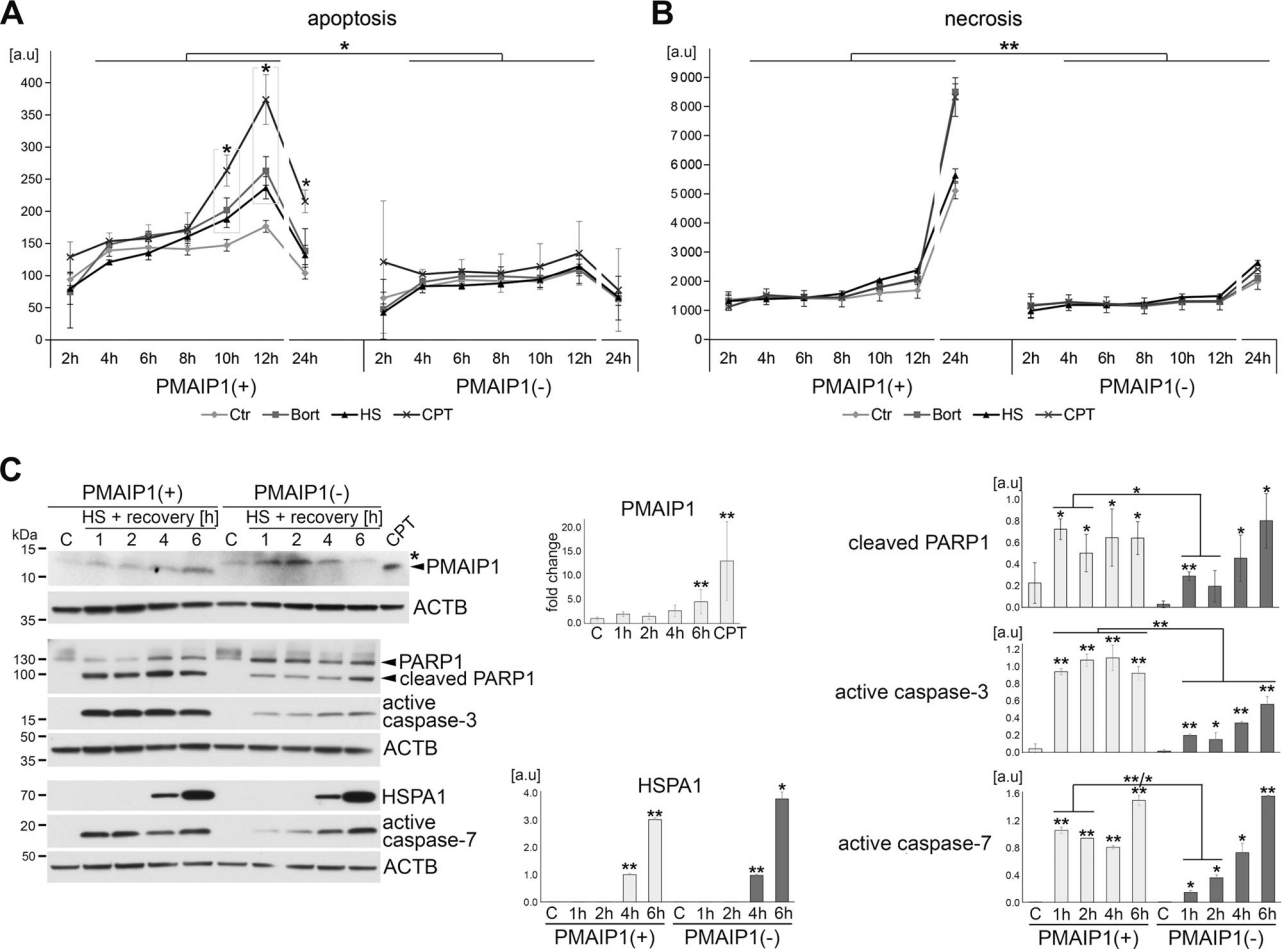
PMAIP1 deficiency reduces heat-induced death and delays caspase-3 and caspase-7 activation. **a** Apoptosis and **b** necrosis of PMAIP1(+) and PMAIP1(−) HECa10 cells monitored between 2 and 24 h after heat shock or during bortezomib (Bort) or camptothecin (CPT) treatments. Shown are mean values ± SD from one (representative) of three independent experiments; the statistically significant difference between treated and untreated samples or PMAIP1(+) and PMAIP1(−) samples is marked with an asterisk (*p < 0.05, **p < 0.001). **c** PMAIP1(+) and PMAIP1(−) cells were heat-shocked for 1 h at 43 °C and protein extracts were analyzed by western blot up to 6 h of recovery. ACTB was used as a loading control. C untreated cells, CPT camptothecin treatment of wild-type HECa10 cells for 6 h (positive control for PMAIP1 induction); unspecific protein band recognized by anti-PMAIP1 Ab is marked with an asterisk. The graphs show the results of densitometric analyses from three independent experiments; *p < 0.05, **p < 0.001.

To demonstrate the physiological relevance of HSF1-mediated *Pmaip1* induction, we next examined the survival of PMAIP1 knockout HECa10 cells subjected to heat shock. Monitoring of the cell state based on the nonlytic detection of phosphatidylserine on the outer leaflet of the cell membranes reveled significant onset of apoptosis 10–12 h after heat shock (or treatment with bortezomib or CPT) in PMAIP1-proficient cells. In marked contrast, this effect was not observed in PMAIP1 knockouts (Fig. 4a). Furthermore, a significant level of necrotic cells was detected 24 h after treatments in PMAIP1-proficient but not in PMAIP1-depleted cells (Fig. 4b). Moreover, classical Annexin V/propidium iodide staining and flow cytometry analyses indicated that PMAIP1-depleted cells were generally less sensitive to heat-induced damage (percentage of apoptotic and necrotic cells was significantly lower than in PMAIP1-proficient cells) (Supplementary Fig. S7). In addition, we compared the kinetics of the heat-induced activation of effector caspases in PMAIP1-proficient and PMAIP1-depleted cells. It is noteworthy that following heat shock the activation of effector caspase-3 and caspase-7, as well as PARP1 cleavage, were markedly delayed in PMAIP1(−) cells in comparison with PMAIP1(+) cells (Fig. 4c). Thus, we postulate that the PMAIP1 upregulation by HSF1 observed during the first hours after treatment is the major reason for heat-induced cell death.

## Discussion

In this study, we described an increased *Pmaip1*/*PMAIP1* gene expression in mouse and human cells which resulted from activation and binding of HSF1 to the *Pmaip1*/*PMAIP1* regulatory sequences in response to heat shock. Moreover, the binding of HSF1 to the *Pmaip1* regulatory sequences and upregulation of the *Pmaip1* expression was observed in cells treated with bortezomib, which suggested a more general mechanism of response to the proteotoxic stress. More interestingly, HSF1 binding was detected not in the promoter, but in the introns of the mouse *Pmaip1* gene. Furthermore, although the human *PMAIP1* gene lacks a perfect HSE consensus sequence, HSF1 also exhibited increased binding to the HSE-like motif located in the intron in response to heat shock concomitant with upregulation of the gene expression. Transcription activation is usually associated with the binding of HSF1 to target gene promoters, although there are also reports showing the involvement of HSF1 in transcription regulation through intronic sequences [39–41]. This mode of regulation could rely on chromatin looping between intronic HSE and other regulatory sequences (e.g., enhancers) as it was shown by Grossi et al. [40]. This may also be the case in the *Pmaip1* regulation. We have noticed that changes in HSE in the second intron also affected the binding of HSF1 in the first intron. Moreover, though human *PMAIP1* contained only the HSE-like sequence in the intron, another HSF1 binding site about 34 kb upstream of the gene was found in the ChIP-Seq data (GSE60984 and GSE137558). More importantly, although HSF1 is considered as a cytoprotective factor, it activates strictly proapoptotic gene which could be the main trigger of heat-induced cell death. The *Pmaip1* upregulation in different mouse organs after heat shock correlates well with their heat sensitivity.

PMAIP1 has well documented proapoptotic activity. Especially in cells exposed to DNA-damaging agents, it is an essential mediator of p53-dependent cell death [42]. It has been previously shown that PMAIP1 is also involved in apoptosis induced by heat shock. PMAIP1 accumulated after heat shock in human lymphoid cells which correlated with MCL1 degradation and BAX activation leading to apoptosis. This effect was inhibited by *PMAIP1* knockdown [43]. Upregulation of the *PMAIP1* gene following heat shock has been documented in several human cell lines [44]. Here we additionally documented the *Pmaip1* upregulation by the elevated temperature in mouse heat-sensitive organs: testes, spleen, thymus, stomach, intestine, but not in liver which is the most heat-resistant organ [45]. In addition, we showed that the heat-induced *PMAIP1* upregulation was not dependent on p53 in human cell lines.

Nakai et al. [46] had already observed that HSF1 could be an apoptosis mediator in mouse testes. Activation of HSF1 induced caspase-3-dependent apoptosis in spermatocytes and such cells were actively eliminated [28, 33, 38, 46, 47]. Consequently, apoptosis of spermatocytes was markedly inhibited in testes of HSF1-null mice exposed to heat shock [48]. It was previously proposed that PHLDA1, which was also identified as a direct target of HSF1, could play a role in promoting heat shock-induced cell death. In fact, heat-induced cell death was to some extent diminished in PHLDA1-null mice testes [49]. However, PHLDA1 is barely detected in spermatogenic cells and neither heat shock nor expression of constitutively active HSF1 results in its accumulation, although both conditions induce massive apoptosis of spermatocytes [50]. To date, several reports demonstrate that PHLDA1 may have both pro- [49] and antiapoptotic [51, 52] functions and the exact role of its expression in apoptotic cell death remains controversial. Nevertheless, in addition to PMAIP1, alternative HSF1-dependent mechanisms of elimination of heat-sensitive cells could be induced by the proteotoxic stress.

One could hypothesize that the choice between pro-survival and pro-death signaling may depend on the ratio among key components induced by HSF1: PMAIP1 and HSPs. It is known that pro-death activity of PMAIP1 can be reduced by HSPs. For example, HSP70 overexpression was connected with a lower degree of *PMAIP1* activation after heat shock and inhibition of apoptosis [43, 44]. It was also postulated that HSP70 overexpression had an indirect influence on *PMAIP1* mRNA stability through a protective effect on *MIR23A* (which could directly bind to the 3′UTR of the *PMAIP1* mRNA) [44]. However, a binding site for *Mir23a* is not present in the mouse *Pmaip1* mRNA, thus this mode of regulation is unlikely in rodents. Apoptotic function of PMAIP1 could be also suppressed by phosphorylation on Ser-13 executed by CDK5 [14] which is also indirectly dependent on HSP70 level. Heat shock caused CDK5 insolubilization and inhibition of its activity which was prevented in cells overexpressing HSP70 [53]. Therefore, to activate pro-death signaling, concurrently with PMAIP1 induction, HSPs expression should be repressed. Indeed, in spermatocytes, which are extremely heat-sensitive, inducible *Hsp* genes expression is blocked following heat shock [29]. The testis-specific variant of HSP70 (HSPA2) is also depleted in this condition [33]. Global gene expression analyses (available on Gene Expression Omnibus, accession no. GSE128996) revealed that *Pmaip1* was the most induced gene in transgenic mice testes overexpressing constitutively active HSF1 specifically in spermatocytes, in which apoptosis was induced [28]. Here we further documented the role of HSF1-regulated PMAIP1 in heat sensitivity of spermatogenic cells. Hence, we postulate that the lack of HSPs (especially HSP70) in connection with HSF1-mediated activation of *Pmaip1* may be the primary cause of cell death in heat-sensitive cells.

## Conclusions

To the best of our knowledge we present here the first evidence that HSF1, a major transcriptional regulator of the HSR, binds to and activates expression of a strictly proapoptotic gene. Thus, we elucidate how HSF1 plays a dual role in response to heat shock, either cytoprotective or lethal. We hypothesize that increased transcription mediated by binding of HSF1 to the *Pmaip1* gene contributes to differential heat/stress sensitivity of cells. HSF1-mediated activation of PMAIP1 in combination with differential expression of cytoprotective HSPs could be the main molecular mechanism involved in switching from pro-survival to pro-death signaling in cells subjected to elevated temperatures.

## Supplementary information

Supplemental Table 1

Supplemental Table 2

Supplemental Figure Legends

Supplemental Figure 1

Supplemental Figure 2

Supplemental Figure 3

Supplemental Figure 4

Supplemental Figure 5

Supplemental Figure 6

Supplemental Figure 7
