# Supplementary material for: Pro-death signaling of cytoprotective heat shock factor 1: upregulation of NOXA leading to apoptosis in heat-sensitive cells
Source: Cell Death Differ. 2020 Jan 29;27(7):2280–92. doi: 10.1038/s41418-020-0501-8 (PMC7308270; doi:10.1038/s41418-020-0501-8)
Supplement: Supplementary file 1 — Supplemental Table 1 [file 41418_2020_501_MOESM1_ESM.docx]

**Table S1**. Characteristics of primers used in PCR analyses

| 1. ChIP-PCR analyses | | | | | |  |
| --- | --- | --- | --- | --- | --- | --- |
| **Gene symbol (Entrez Gene ID)** | **HSEs/HSE-like position**^a^ | **Primers sequences** | **Product lenght [bp]** | **Position of the amplified sequence**^a^ | |  |
| *Pmaip1* (58801) mouse | (+287) – (+311)  (+350) – (+369) | F: GCAGGGATGGGGTTGAGGTCAT  R: TCCCAATGATACGCTGGACTGGA | 189 | (+241) – (+429)  first intron | |  |
|  | (+2488) – (+2502) | F: ATGAGGAGCCCAAGCCCAAC  R: ACCCCTGTGGAGGTGAGCAA | 201 | (+2362) – (+2562)  second intron | |  |
|  |  | F: GCGCGTGCCTTAGGAACACTGTA  R: TGGAGGTGAGCAACCTGCAGAGA | 91 | (+2465) – (+2555)  second intron | |  |
| *PMAIP1* (5366) human | (-16) – (-2)  (+110) – (+129) | F: GCGCGGGGATCTCAGAGTTT  R: CGTTCTTGCGCGCCTTCTTC | 223 | (+24) – (+245)  first exon | |  |
|  | (+432) – (+451)  (+450) – (464) | F: GCGGAGATGCCTGGGAAGAA  R: CTTGGTCCCTCGCCGTAC | 292 | (+213) – (+504)  intron | |  |
|  |  | F: ACAGACTGCACGGGGGTCAA  R: GCTGCATCCCAATCGCAAAT | 153 | (+376) – (+528)  intron | |  |
| *Hsph1* (15505) mouse | (-55) – (-36)  (+10) – (+29) | F: CTGTCACCATGGCAACTCAG  R: CCAATCGCTCAGCCTTATGT | 175 | (-114) – (+61)  promoter | |  |
| *HSPH1* (10808) human | (-64) – (-45)  (+12) – (+31) | F: TGCCCATTGGGTAGAATCTTTC  R: GAGGTCCCACTTCCTCAGCCTTA | 151 | (-82) – (+69)  promoter | |  |
| *HSPA1A* (3303) human | (-88) – (-64) | F: cggcactctggcctctgatt  R: gacccgccttttcccttctg | 128 | (-141) – (-14)  promoter | |  |
| Negative locus (NC_000012.12) |  | F: ATGGTTGCCACTGGGGATCT  R: TGCCAAAGCCTAGGGGAAGA | 174 |  | |  |
| 1. RT-PCR analyses | | | | | |  |
| **Gene symbol** | **NCBI reference sequence** | **Primers sequences** | **Product lenght [bp]** | | **Position in the reference sequence (comments)** | |
| *Pmaip1* | NM_021451.2 | F: cctactgaagctcggtgcgtct  R: tgcgaactcaggtggtagctc | 209 | | 21 – 229 (5’ end) | |
|  |  | F: atgaggagcccaagcccaac  R: ccatcaaccggcggaactt | 198 | | 332 – 529 (3’ end) | |
| *PMAIP1* | NM_021127.2 | F: gcggagatgcctgggaagaa  R: tgatgaaacgtgcacctcctga | 238 | | 213 – 450 | |
|  |  | F: ggagacaaactgaacttccggca  R: ggcacccatgaatgcaccttca | 194 | | 315 – 508  (for qPCR) | |
| *Hspa1a*  *Hspa1b* | NM_010479.2  NM_010478.2 | F: ccatccagagacaagcgaag  R: cgtttagaccggcgatcac | 699 | | 38 – 736 | |
|  |  | F: acaagagaagcagagcgagc  R: atcgccgtgttcttggccat | 194 | | 58 – 251  (for qPCR) | |
| *HSPA1A*  *HSPA1B* | NM_005345.5  NM_005346.4 | F: cgccgtttccagcccccagtc  R: cgttgagccccgcgatcaca | 557 | | 191 – 748  164 – 721 | |
| *HSPA1A* | NM_005345.5 | F: ccgagaaggacgagtttgag  R: aacagcaatcttggaaaggc | 189 | | 2003 – 2191  (for qPCR) | |
| *HSF1* | NM_005526.2 | F: ccagcaacagaaagtcgtca  R: gagctcattcttgtccaggc | 325 in mutant *HSF1* | | 710 – 1322 | |
| *Hnrnpk* | NM_001301341.1 | F: tgggttcagtgctgatgaaa  R: aataggtccgccaagatcac | 151 | | 1285 – 1435 | |
| *HNRNPK* | NM_002140.4 | F: atgctgtcctcattccactgac  R: cgcgacggtcatcaaacatca | 194 | | 936 – 1129 | |
| *Gapdh* | NM_008084.3 | F: tggtgaagcaggcatctgagg  R: catgaggtccaccaccctgt | 203 | | 1016 - 1218 | |
| *GAPDH* | NM_002046.6 | F: tggtgaagcaggcgtcggagg  R: catgaggtccaccaccctgt | 203 | | 858 - 1060 | |
| *Actb* | NM_007393 | F: ggacttcgagcaagagatgg  R: agcactgtgttggcgtacag | 234 | | 742 – 975 | |
| *Rn18s (18s rRNA)*  *RNA18S5* | NR_003278.3  NR_003286.2 | F: gttggtggagcgatttgtctgg  R: gcagccccggatctaagg | 178 | | 1345 – 1522  1345 – 1521 | |

^a^against transcription start site (+1) according to DBTSS (<http://dbtss.hgc.jp/>) and RefSeq
