# Supplementary material for: Pro-death signaling of cytoprotective heat shock factor 1: upregulation of NOXA leading to apoptosis in heat-sensitive cells
Source: Cell Death Differ. 2020 Jan 29;27(7):2280–92. doi: 10.1038/s41418-020-0501-8 (PMC7308270; doi:10.1038/s41418-020-0501-8)
Supplement: Supplementary file 2 — Supplemental Table 2 [file 41418_2020_501_MOESM2_ESM.docx]

**Table S2**. Edit-R CRISPR RNA (crRNA) sequences targeting the mouse *Pmaip1* or the human *HSF1* genes

|  | *Pmaip1* targeting sequence | position |  | strand |
| --- | --- | --- | --- | --- |
| 1. | 5’-GAGTGCACCGGACATAACTG-3’ | chr18:66460904-66460926 | Exon 2 | + |
| 2. | 5’-TTCCGACGCGCCTTTCTCCC-3’ | chr18:66458764-66458786 | Exon 1 | - |
| 3. | 5’-TACCTGCTGGCACCCGGGTT-3’ | chr18:66460979-66461001 | Exon 2 | - |
|  | *HSF1* targeting sequence |  |  |  |
| 1. | 5’-GGTGTCCGGGTCGCTCACGA-3’ | chr8:144291850-144291831 | Exon 1 | - |
| 2. | 5’-AAAGTGGTCCACATCGAGCA-3’ | chr8:144309466-144309485 | Exon 3 | + |
