# Supplementary material for: Pro-death signaling of cytoprotective heat shock factor 1: upregulation of NOXA leading to apoptosis in heat-sensitive cells
Source: Cell Death Differ. 2020 Jan 29;27(7):2280–92. doi: 10.1038/s41418-020-0501-8 (PMC7308270; doi:10.1038/s41418-020-0501-8)
Supplement: Supplementary file 3 — Supplemental Figure Legends [file 41418_2020_501_MOESM3_ESM.docx]

**SUPPLEMENTAL FIGURE LEGENDS**

**Fig. S1. Specificity of the anti-PMAIP1 antibody. (A)** Mouse NIH3T3 cells were transiently transfected with the vector coding for PMAIP1/EGFP fusion protein (+) or control vector (-). Anti-PMAIP1 antibody (PRS2437, Merck KGaA) can recognize the fusion protein (arrowhead, right part of the blot) which is inhibited by blocking peptide (SBP2437, Merck KGaA) (left part). (**B**) Anti-PMAIP1 antibody and the blocking peptide were tested in HECa10 cells: wild-type (WT), control clones obtained after introducing non-targeting control crRNA (PMAIP1(+)) or in clones with knocked-out *Pmaip1* gene using the gene-specific crRNAs (for CRISPR/Cas9 mediated genome editing) (PMAIP1(-)). Cells were either untreated or PMAIP1 expression was induced by heat shock (HS, 1 h at 43°C and 6 h of recovery) or camptothecin (CPT) treatment for 6 h. Arrowheads show the position of the PMAIP1 protein bands of the expected size (~11-12 kDa). The protein band of ~17kDa preferentially recognized by the antibody (marked with an asterisk), although inhibited by blocking peptide (lower panel), it is still present in PMAIP1(-) cells. Therefore, this protein should not be considered as PMAIP1. (**C**) The 17 kDa protein band recognized by the anti-PMAIP1 antibody is very abundant in mouse stomach and can be readily detected in 5-10 μg of protein extracts, which makes barely possible the detection of PMAIP1 protein of expected size (11-12 kDa). In peptide competition assay the antibody was pre-incubated for 1 h at room temperature with a 7.5-times excess of the synthetic peptide antigen. ACTB was used as a loading control.

**Fig. S2.** **Kinetics of PMAIP1 and apoptosis induction in heat-shocked mouse testes.** Detection of PMAIP1 by immunofluorescence (green) and apoptotic DNA breaks (by TUNEL assay, red; DNA stained with DAPI, blue) in seminiferous tubules (stages IX-X) of untreated mice and after heat shock *in vivo* at 43°C. Scale bar – 50 µm.

**Fig. S3. PMAIP1 is activated by heat shock in heat-sensitive mouse tissues.** (**A**) Induction of *Pmaip1* transcription assayed by RT-PCR in selected organs of mice after heat shock (HS) performed *in vivo* at 43°C. *Hspa1* was used as a positive control for the heat shock response, *Gapdh*, *Actb*, *18S rRNA* – as a loading controls. C, control, physiological temperature. (**B**) Mouse PMAIP1 of the calculated size (11.5 kDa) can be detected by western blot after heat shock in the spleen and stomach, but is hardly detected in the kidney. Human PMAIP1 of the calculated size (6 kDa) was detected in A549 cells after CPT or A+N treatment. Black arrowheads indicate protein bands corresponding to theoretically calculated molecular mass of PMAIP1, white arrowheads – protein bands in the mouse stomach putatively corresponding to size of human PMAIP1, asterisks – unspecific bands ~17 kDa (see Fig. S1). In case of stomach, 10 µg (upper blot) or 30 µg (lower blot) total protein extracts were loaded. ACTB and HSPA1 were used as controls for loading and the heat shock response, respectively. M1, M2, markers; A+N, 5 nM actinomycin D and 5μM nutlin-3a for 48 h; CPT, camptothecin treatment; C, untreated control (for TUNEL tests in heat shocked mouse organs see Fig. S4).

**Fig. S4.** **Heat-sensitivity of different mouse tissues.** Detection of apoptotic DNA breaks (by TUNEL assay, red; DNA stained with DAPI, blue) in organs of untreated mice and after heat shock *in vivo* for 30 min at 43°C and indicated time of recovery­. A negative control was incubated only with Label Solution without terminal transferase. Scale bars – 50 or 100 µm.

**Fig. S5. Heat shock-induced activation of *Pmaip1* in different cell lines *in vitro*.** (**A**) Heat shock-induced activation of *Pmaip1* in mouse cell lines originating from endothelial cells of peripheral lymph nodes (HECa10), melanoma (B16-F10), renal adenocarcinoma (Renca), embryo fibroblasts (NIH3T3), colon adenocarcinoma (C26), and lung carcinoma (LL2); cells were heat shocked for one hour at 43°C and recovered for two hours, then analyzed by RT-PCR. (**B**) The analysis of *Pmaip1*/*PMAIP1* expression up to 6 h of recovery in mouse HECa10 and B16-F10 cells and in human 1205Lu cells. *Hspa1* (*Hspa1a* and *Hspa1b*) was used as a positive control for the heat shock response, *Gapdh* and *18S rRNA* – as loading controls. C, control, physiological temperature, HS, heat shock. (**C**) Percentage of dead HECa10 or B16-F10 cells (quantified on a FACS Canto cytometer after propidium iodide staining) is shown as signals and as differences between HS (heat shock for one hour at 45°C with subsequent recovery for 17 h) and C (untreated control, culture at 37°C). Shown are the mean values ±SD from four experiments. *p < 0.05. (**D**) HSF1 binding in *Pmaip1*/*PMAIP1* introns analyzed by ChIP-PCR in mouse HECa10 and B16-F10 cells and human 1205Lu cells, untreated or 15 min after heat shock at 43°C. Binding to the *Hsph1*/*HSPH1* promoter served as a positive control. C, control, physiological temperature; HS, heat shock; M, marker; – +, negative and positive PCR controls.

**Fig. S6. *Pmaip1* is up-regulated in HECa10 cells by bortezomib and sodium arsenite, known inducers of HSF1.** (**A**) Sensitivity of HECa10 cells to bortezomib estimated by the MTT assay. C – untreated control; D – solvent (DMSO) control. Activation of HSF1 **(B)** and induction of HSPA1 **(C)** by different concentrations of bortezomib in HECa10 cells. **(D)** Assessment of the HSF1 binding to HSE sequences present in introns of *Pmaip1* and promoter of *Hsph1* genes by 32 nM bortezomib in HECa10 and B16-F10 cells. **(E)** Induction of *Pmaip1* and *Hspa1* gene expression by 32 nM bortezomib in HECa10 and B16-F10 cells. **(F)** Induction of *Pmaip1* and *Hspa1* gene expression by 100 µM sodium arsenite in HECa10 and B16-F10 cells.

**Fig. S7. The sensitivity of PMAIP1-proficient and PMAIP1-deficient cells to heat shock.** PMAIP1(+) and PMAIP1(-) HECa10 cells were heat-shocked for one hour at 43°C, stained with FITC Annexin V Apoptosis Detection Kit with 7-AAD (7-Aminoactinomycin D) (Cat. No. 640922) after indicated recovery time, and analyzed on a FACS Canto cytometer (Becton Dickinson) to count the number of viable and apoptotic cells. Box-plots represent quantification of the results; shown are lower quartile, median, upper quartile (boxes), as well as minimum and maximum values (whiskers). *p < 0.05; **p < 0.001. The gating of a representative cell distribution (Q1-Q4) is shown below.
