# Supplementary figures and images for: Pro-death signaling of cytoprotective heat shock factor 1: upregulation of NOXA leading to apoptosis in heat-sensitive cells
Source: Cell Death Differ. 2020 Jan 29;27(7):2280–92. doi: 10.1038/s41418-020-0501-8 (PMC7308270; doi:10.1038/s41418-020-0501-8)

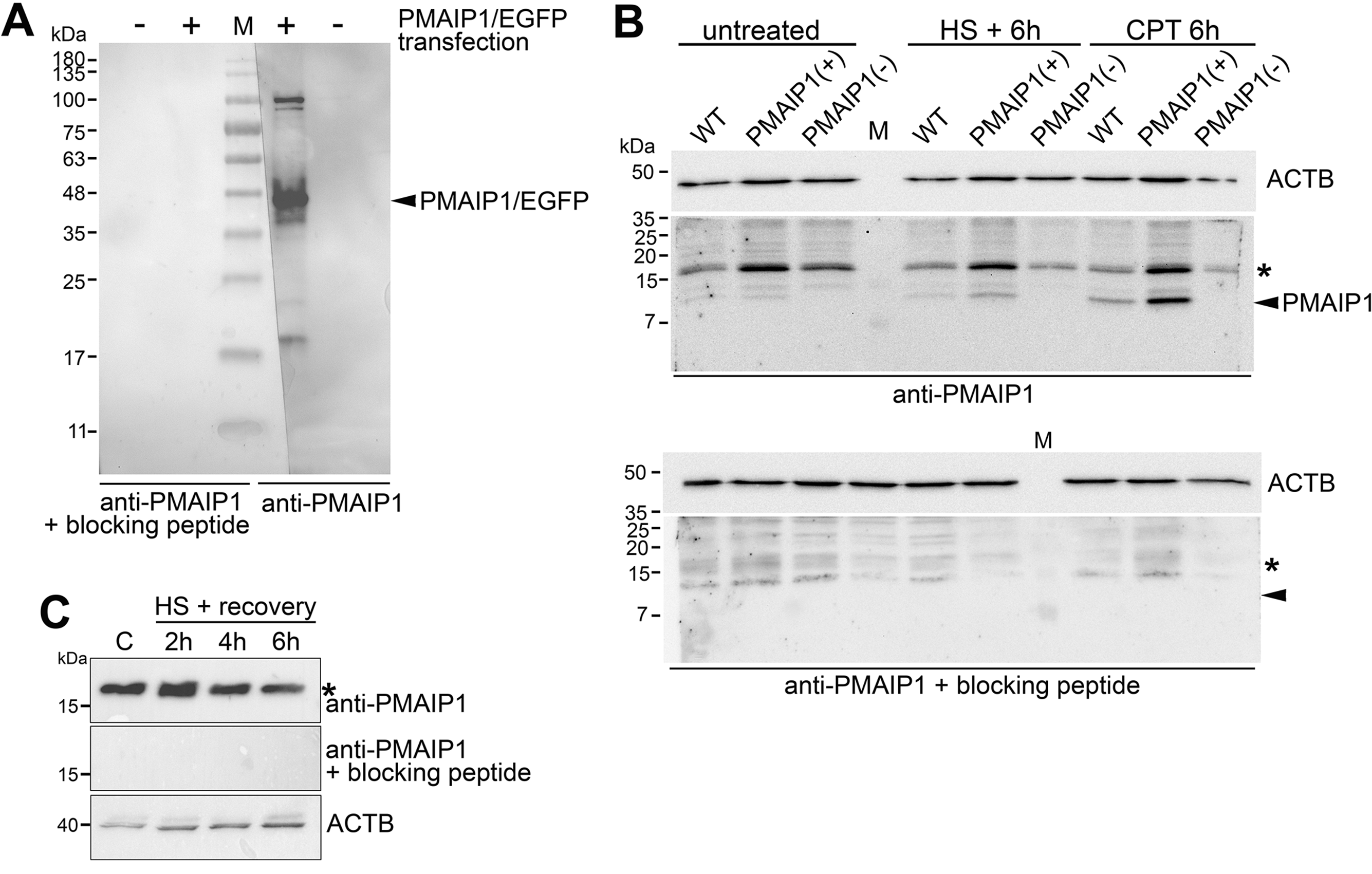

Supplement: Supplementary file 4 — Supplemental Figure 1 [file 41418_2020_501_MOESM4_ESM.tif]

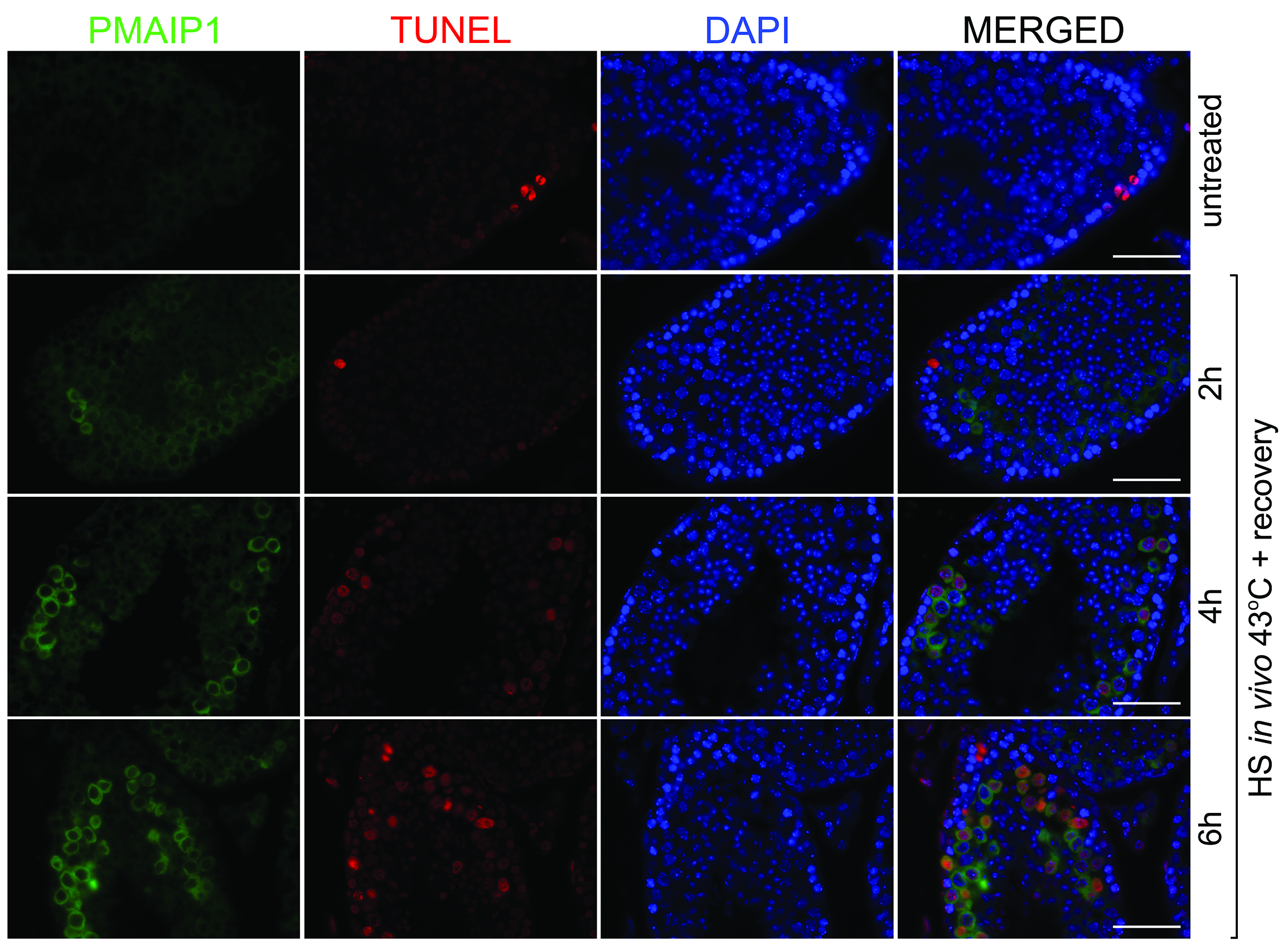

Supplement: Supplementary file 5 — Supplemental Figure 2 [file 41418_2020_501_MOESM5_ESM.tif]

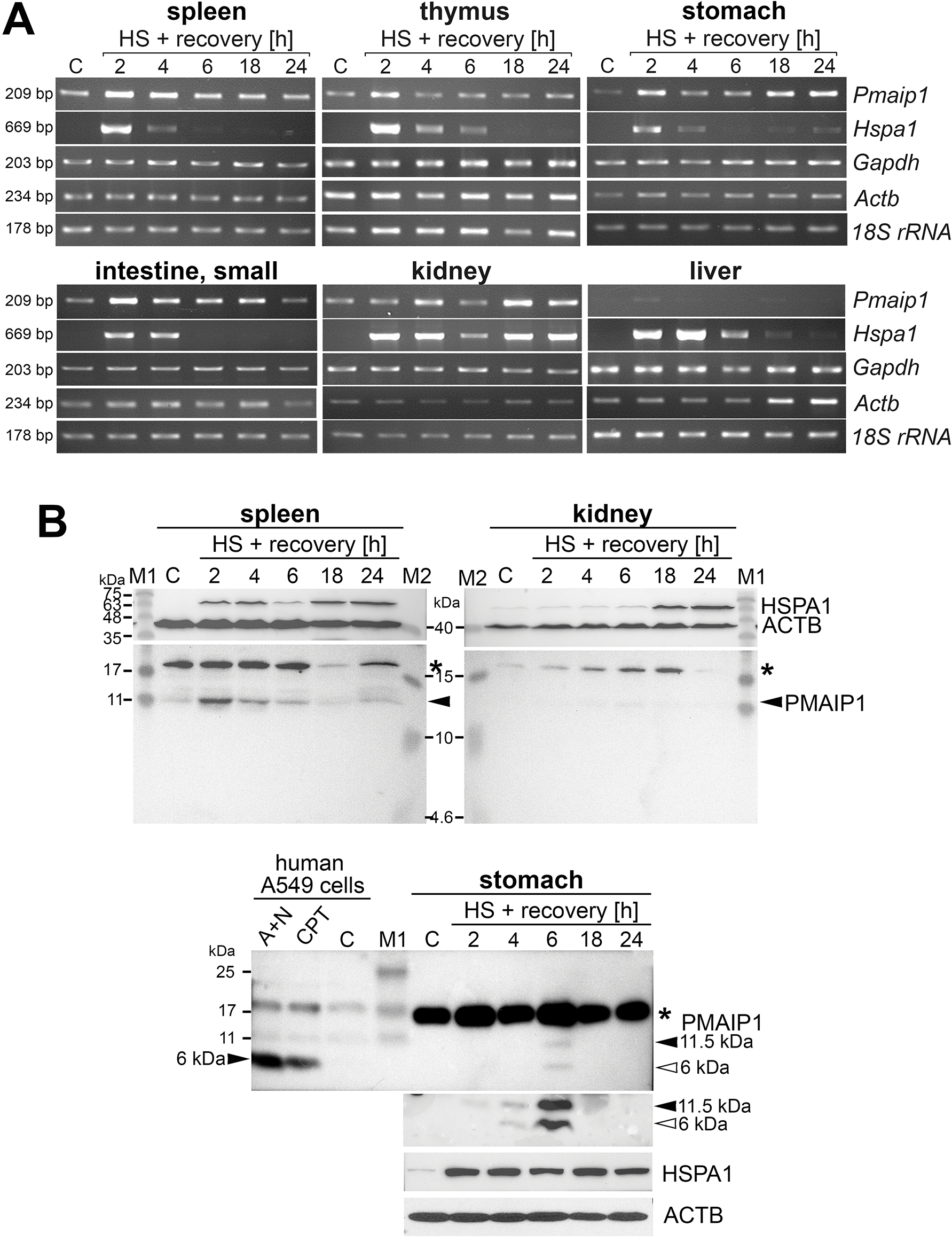

Supplement: Supplementary file 6 — Supplemental Figure 3 [file 41418_2020_501_MOESM6_ESM.tif]

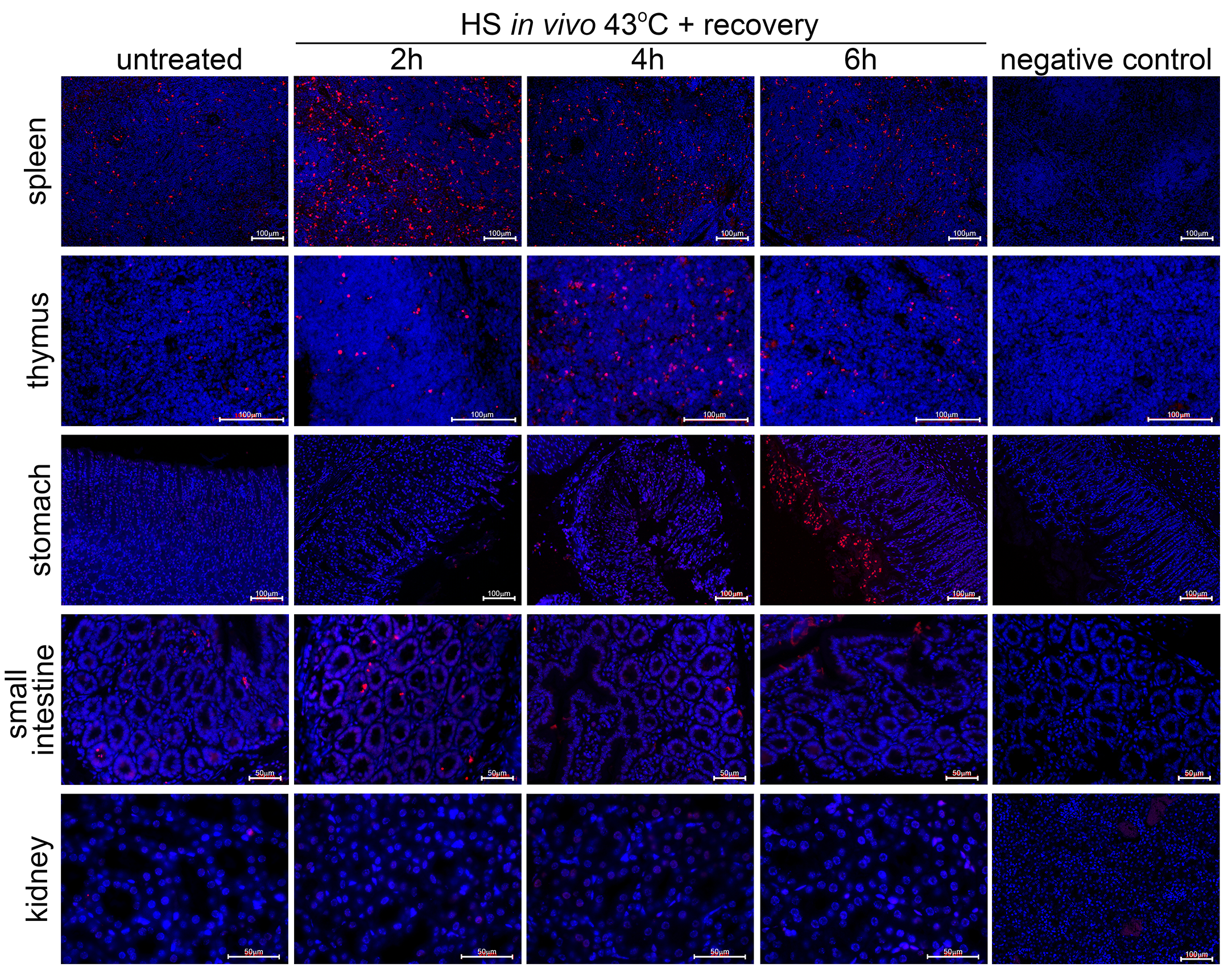

Supplement: Supplementary file 7 — Supplemental Figure 4 [file 41418_2020_501_MOESM7_ESM.tif]

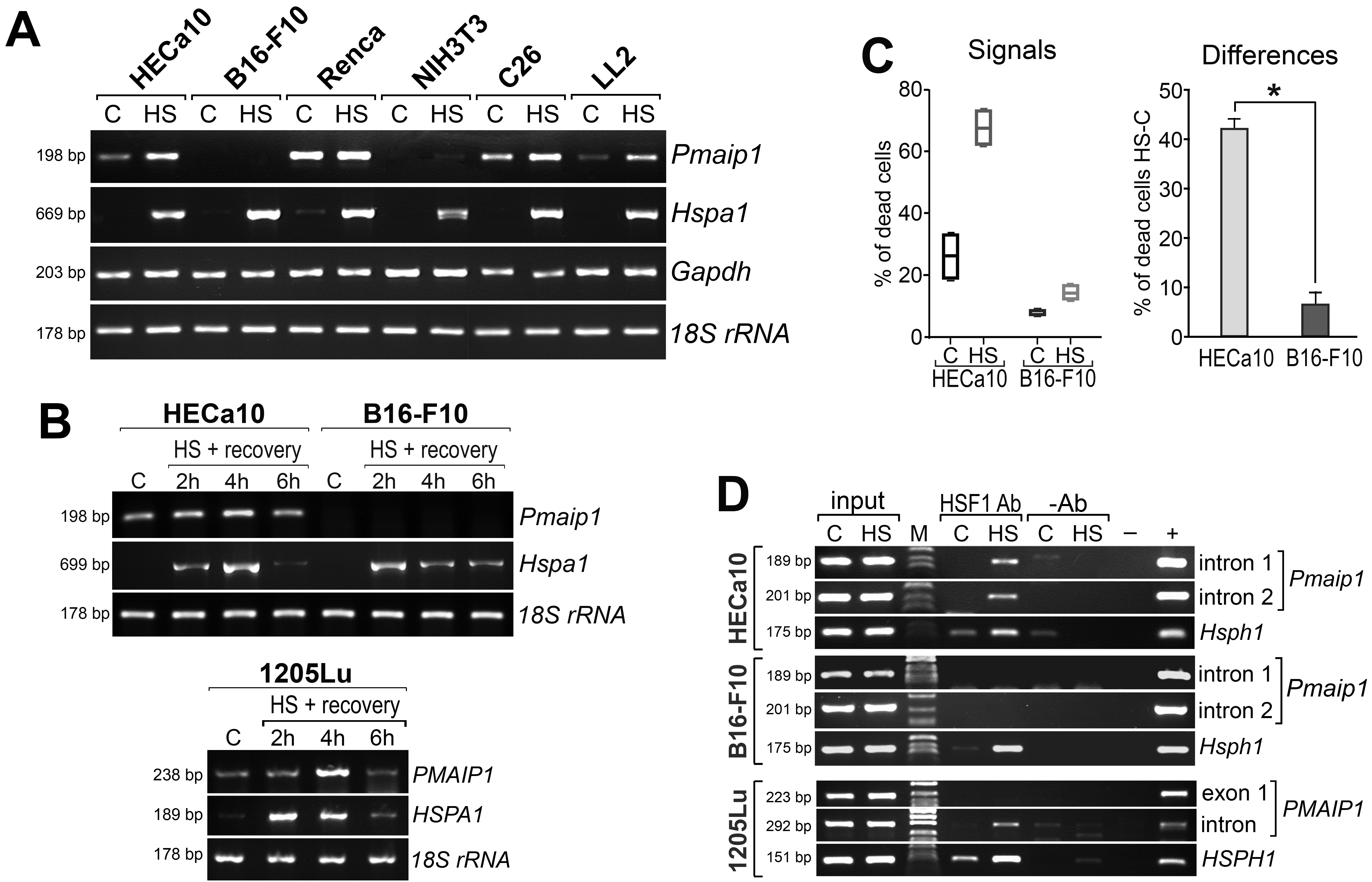

Supplement: Supplementary file 8 — Supplemental Figure 5 [file 41418_2020_501_MOESM8_ESM.tif]

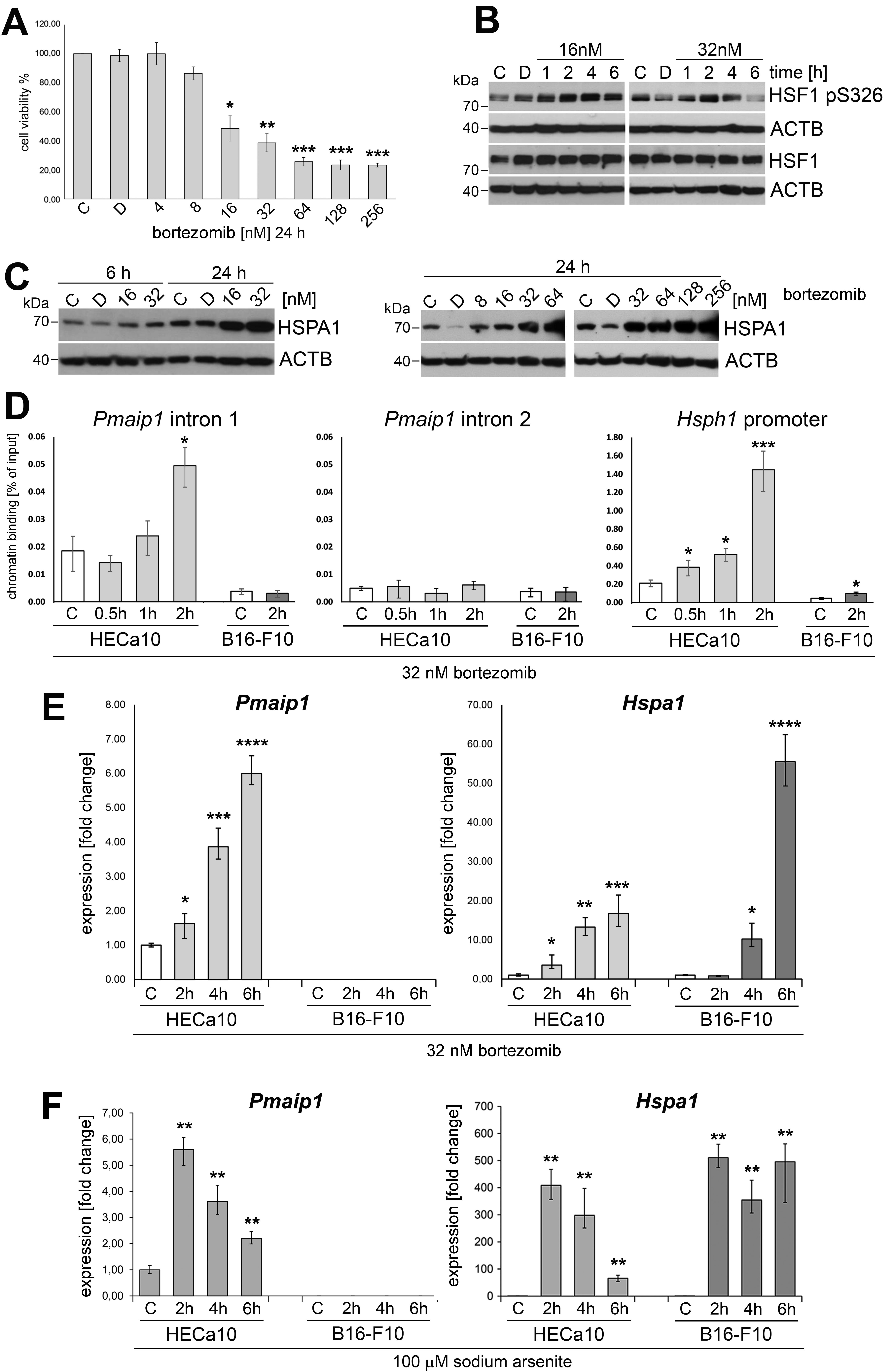

Supplement: Supplementary file 9 — Supplemental Figure 6 [file 41418_2020_501_MOESM9_ESM.tif]

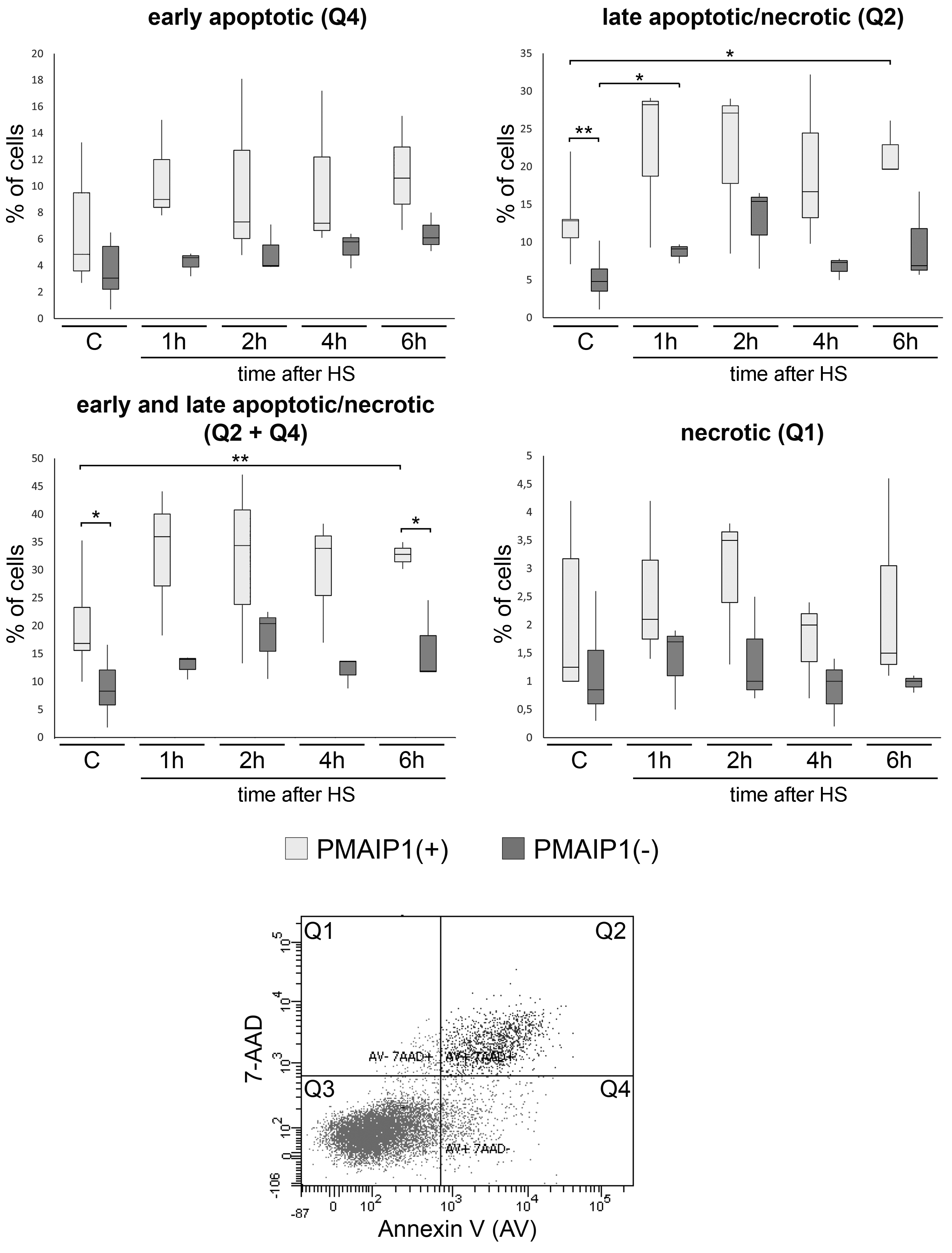

Supplement: Supplementary file 10 — Supplemental Figure 7 [file 41418_2020_501_MOESM10_ESM.tif]
